# Placental Lactogen as a Marker of Maternal Obesity, Diabetes, and Fetal Growth Abnormalities: Current Knowledge and Clinical Perspectives

**DOI:** 10.3390/jcm9041142

**Published:** 2020-04-16

**Authors:** Rafał Sibiak, Maurycy Jankowski, Paweł Gutaj, Paul Mozdziak, Bartosz Kempisty, Ewa Wender-Ożegowska

**Affiliations:** 1 Division of Reproduction, Department of Obstetrics, Gynecology, and Gynecologic Oncology, Poznań University of Medical Sciences, 33 Polna St, 60-535 Poznan, Poland; 75094@student.ump.edu.pl (R.S.); pawelgutaj@ump.edu.pl (P.G.); 2 Department of Anatomy, Poznań University of Medical Sciences, 6 Święcickiego St, 60-781 Poznan, Poland; mjankowski@ump.edu.pl (M.J.); bkempisty@ump.edu.pl (B.K.); 3 Physiology Graduate Program, North Carolina State University, Campus Box 7608, Raleigh, NC 27695-7608, USA; pemozdzi@ncsu.edu; 4 Department of Histology and Embryology, Poznań University of Medical Sciences, 6 Święcickiego St, 60-781 Poznan, Poland; 5 Department of Obstetrics and Gynecology, University Hospital and Masaryk University, 20 Jihlavská St., 625 00 Brno, Czech Republic; 6 Department of Veterinary Surgery, Institute of Veterinary Medicine, Nicolaus Copernicus University in Toruń, 87-100 Toruń, Poland

**Keywords:** diabetes, fetal growth, placental lactogen, obesity

## Abstract

Placental lactogen (PL) is a peptide hormone secreted throughout pregnancy by both animal and human specialized endocrine cells. PL plays an important role in the regulation of insulin secretion in pancreatic β-cells, stimulating their proliferation and promoting the expression of anti-apoptotic proteins. Cases of pregnancy affected by metabolic conditions, including obesity and diabetes, are related to alterations in the PL secretion pattern. Whereas obesity is most often associated with lower PL serum concentrations, diabetes results in increased PL blood levels. Disruptions in PL secretion are thought to be associated with an increased prevalence of gestational complications, such as placental dysfunction, diabetic retinopathy, and abnormalities in fetal growth. PL is believed to be positively correlated with birth weight. The impaired regulation of PL secretion could contribute to an increased incidence of both growth retardation and fetal macrosomia. Moreover, the dysregulation of PL production during the intrauterine period could affect the metabolic status in adulthood. PL concentration measurement could be useful in the prediction of fetal macrosomia in women with normal oral glucose tolerance test (OGTT) results or in evaluating the risk of fetal growth restriction, but its application in standard clinical practice seems to be limited in the era of ultrasonography.

## 1. Introduction

According to the information provided by the Centers for Disease Control and Prevention, 36.5% of women aged 20–39 in the United States suffer from obesity [1], which is connected with a variety of other obesity-related diseases, such as metabolic syndrome, hypertension, type 2 diabetes, dyslipidemias, atherosclerosis, and several cancers [2,3]. Furthermore, obesity and its consequences are associated with an increased prevalence of conditions such as pre-eclampsia, gestational diabetes (GDM), and fetal macrosomia, which can lead to severe gestational complications and an elevated risk of miscarriage or stillbirth [4,5,6].

Diabetes remains the most important causative factor for serious perinatal complications, including various congenital malformations, preeclampsia, and fetal macrosomia [7,8,9]. Even though the implementation of intensive insulin therapy significantly reduces the risk of development of the pathologies mentioned above, patients with well-controlled glucose metabolism nevertheless struggle with negative consequences of diabetes [10,11]. Therefore, a better understanding of other molecular aspects of fetal intrauterine growth regulation could become the basis of perinatal outcome improvement therapies.

Placental lactogen (PL), also known as chorionic somatotropin, is a peptide hormone produced during pregnancy, in humans and other animals, by specialized endocrine cells. More specifically, PL is synthesized by syncytiotrophoblast cells in humans, trophoblast giant cells in rats and mice, and trophoblastic binucleate cells in cows and sheep—only these species are included in this review [12,13,14,15,16,17]. PL is classified as a member of the somatotropin family, which also includes growth hormone (GH), prolactin (PRL), and placental growth hormone (GH-V), mainly due to the similarities observed in their molecular structure [12,18,19,20]. More details about the PL family genes and encoded proteins have been described by Handwerger et al. in their excellent review [21]. In humans, PL mainly binds to prolactin receptors and with a much lower affinity to growth hormone receptors [21]. Moreover, specific PL receptors have been found in human fetal skeletal muscles [22]. Similar to humans, PL in ruminants binds with a high affinity to PRL receptors, but it also has a high affinity to GH receptors [17]. Mouse PL has a higher affinity to PRL receptors than GH receptors; however, in contrast to humans, in mice and rats, there are two types of active PL with distinct biological activity [19,23,24,25]. Due to the described differences, the results of studies performed in other species cannot be directly extrapolated to humans. PL is detectable in both umbilical cord blood samples and maternal blood from the first trimester of physiological pregnancy, and its concentrations increase in the later stages of fetal and placental development [18,21,26]. Although the PL expression is only present in placental tissue cells, it is considered to play a significant role in the regulation of both maternal and fetal metabolic adaptations throughout the pregnancy [19,27]. The secretion of PL, as well as other placental hormones, could promote the state of systemic insulin resistance and subsequently be responsible for the elevation of maternal blood glucose levels to facilitate the supply of energetic substrates to the fetus [28,29,30].

## 2. Scope and Methods

As PL is considered to be important for the regulation of fetal development and is a clinical biomarker of the maternal and fetal metabolic status, this review summarizes the current knowledge on its involvement in obesity, diabetes, and fetal growth abnormalities, and outlines the perspectives associated with its clinical use as an additional screening tool for the detection of GDM and its possible consequences in the later stages of pregnancy.

This review was compiled by our study group to gather and systematize the current state of knowledge on PL before the commencement of a series of clinical studies focused on PL in our research facility. PubMed and Scopus databases were searched for relevant references from the first records until December 2019, using the following terms: “placental lactogen diabetes”, “placental lactogen obesity”, “placental lactogen glucose intolerance”, “placental lactogen hypoglycemia”, “placental lactogen hyperglycemia”, “placental lactogen insulin resistance”, “placental lactogen metabolic syndrome”, “placental lactogen fetal growth”, “placental lactogen insulin”, “placental lactogen IGF”, “placental lactogen leptin”, “placental lactogen ghrelin”, “placental lactogen neuropeptide Y”, “placental lactogen glycolysis”, “placental lactogen gluconeogenesis”, and “placental lactogen lipolysis”. All of the above-mentioned terms were also searched in connection with the phrase “chorionic somatomammotropin”. Nonetheless, the review material was restricted to articles written in English, which could be regarded as a possible limitation of the study.

## 3. Placental Lactogen in Animal Models (In Vitro and In Vivo Studies)

### 3.1. Impact on Fetal Growth

Baker et al. experimentally induced a decrease in PL during ovine pregnancy and speculated that a significant decrease in PL levels could be associated with an elevated incidence of intrauterine growth restriction (IUGR) in the offspring. The expression of PL was modified in vivo using three different lentiviral-mediated PL-targeted short-hairpin RNA constructs. The most efficient construct reduced both near-term (135 days of gestation) PL mRNA expression (50%) and blood protein concentrations (38%), resulting in a reduction of the placental, fetal, and fetal liver weight (52%, 32%, and 41%, respectively). The PL-targeted constructs also significantly lowered the levels of insulin-like growth factor-1 (IGF1) and IGF2 mRNA in fetal liver tissue. Moreover, fetal IGF1 concentrations in the umbilical artery were markedly reduced compared with the control group [31].

Jeckel et al. studied sheep pregnancy to present the effect of low PL concentrations on fetal development. The authors modified placental gene expression using lentiviral infection of fully expanded ovine blastocysts, as described by Baker et al. [31], resulting in fetuses being exposed to lower PL concentrations. The authors found a significant decrease (41%) in the uterine vein PL concentrations, and the PL mRNA and PL concentrations measured in placental tissue at 50 days of gestation (dGA) were not found to exhibit a significant difference. The described procedure also resulted in significant deficiencies in the fetal weight (21%) and fetal liver weight (21%). In contrast, placental weight reduction (17%), and the expression of *IGF1* and *IGF2* genes in fetal liver tissue at 50 dGA were not found to be significant. Furthermore, the placental *IGF-1* and *IGF-2* mRNA concentrations were measured at 50 and 135 dGA. A significant reduction in *IGF-1* and *IGF-2* mRNA concentrations in placental tissue was only detected at 135 dGA (66% and 53%, respectively) [32].

Furthermore, to examine the possible effects of PL on early organogenesis, Karabulut et al. conducted a study on 9.5 day rat embryos. The embryos were in vitro cultured for 48 h in the presence and absence of PL. Embryos treated with PL solution presented improved parameters of fetal growth. The authors noticed a significant increase in the morphological score, yolk sac diameter, crown-rump length, somite number, and embryonic and yolk sac protein content. In the next step, to test the hypothesis that the described effect of PL on rat embryo development could be mediated by *IGF-1* and *IGF-2*, the embryos were cultured in PL solution supplemented with antisera of both of these proteins. The presence of the abovementioned antibodies resulted in decreased growth parameters in the cultures [33].

These results support the hypothesis that PL could play a key role in the regulation of fetal development, with its deficiency being connected with fetal and placental growth impairments in animal models. Fetal growth abnormalities are directly associated with disrupted *IGF-1* and *IGF-2* expression, normally stimulated by physiological PL concentrations.

### 3.2. Placental Lactogen and Metabolic Changes

To examine the potential influence of PL on perinatal and postnatal growth and metabolic adaptations, Fleenor et al. created a new mouse model (a mouse with a lack of prolactin receptors (*PRLR*) and impaired pituitary growth hormone secretion), for which the parameters were later compared with wild-type mice. On day 7 of life, double-mutant mice had a lower body weight and higher blood glucose concentrations compared with mice with isolated *PRLR* or GH deficiency. During the first weeks of life, double-mutant mice also presented growth retardation, developed hypoglycemia, and exhibited decreased blood levels of both *IGF1* and *IGF2*. During the next months of observation, double-mutant mice additionally developed obesity, hyperleptinemia, fasting hyperglycemia, insulin resistance, and glucose intolerance. Moreover, at ten months of age, double-mutant mice exhibited a higher body fat percentage, increased glucose intolerance, and higher blood leptin concentrations compared to the specimen with isolated *PRLR* expression or GH secretion abnormalities [34]. Based on these findings, we can formulate a thesis that lactogen could play a role in regulating mouse neonatal growth and their future metabolic status, as an expression of its receptors resulted in enhanced growth retardation and a poorer metabolic status compared with mice with isolated GH deficiency. Furthermore, at the age of 12–16 months, double-mutant mice were found to have fasting hyperinsulinemia, hyperamylinemia, hyperleptinemia, and a decreased ratio of adiponectin to leptin. Abnormalities in lactogen receptor expression and GH deficiency not only dysregulated the pancreatic hormone release pattern, but also changed the pattern of adipocytokine production [35].

Several lactogens (PRL, GH, and PL) were suspected of having the ability to increase glucose oxidation in murine adipose tissue, similar to endogenic insulin. Mouse adipose tissue segments from the parametrial fat pads were incubated with the presence of the previously mentioned hormones. To examine their effect on glucose oxidation, a solution of 0.5 μCi/mL d-[U-^14^C] glucose was added to the samples. After 2 h of incubation, ^14^CO_2_ produced by oxidation of the radioactive glucose was collected and counted. Finally, only the mouse growth hormone had a significant positive effect on glucose oxidation in adipose tissue collected from both pregnant and non-pregnant mice [36]. Leturque et al. investigated how PL stimulation could affect glucose metabolism in rat skeletal muscles (soleus, extensor digitorum longus, and epitrochlearis). Ovine PL had no effect on hexose transport, glycogen synthesis, and the glycolysis rate in vitro, both before and after stimulation by insulin [37]. Another study analyzed the influence of PL on adipose tissue in ruminants. The samples of subcutaneous adipose tissue were incubated in the presence of the following hormones: GH, PRL, and PL. To determine their potential lipolytic effect, glycerol concentrations in the samples were assessed after the incubation. The study revealed that PL and other hormones do not affect the rate of lipolysis at any dose [38]. Furthermore, it has been established that PL does not stimulate lipolysis and does not inhibit the glucagon-stimulated lipolysis in chicken adipose tissue [39].

Based on the results of those animal studies, we can conclude that PL does not play a significant role in glucose and lipid metabolism in adult animal tissues [36,37,38,39]. Nevertheless, it should be emphasized that a lack of its receptors and presumably pathological changes in its biological concentrations during pregnancy could contribute to multiple long-term metabolic consequences [34,35].

### 3.3. Role in Pancreatic Beta Cells

Members of the PL family hormones, such as prolactin and PL, are regarded as stimulators of the intensive proliferation of pancreatic β-cells in pregnant rodents. However, the proliferative effect of prolactin on human β-cells in vitro was not as spectacular as that observed in rodents [40]. The possible mechanism of the proliferative effect of endogenic PL on pancreatic islets is closely associated with the stimulation of prolactin receptors in rodent β-cells. Transgenic mice with a specific deletion of *PRLR* from β-cells exhibit reduced β-cell expansion during pregnancy, leading to the development of gestational diabetes [41].

Human studies suggest that lactogens are less effective in the regulation of beta cell adaptations to pregnancy. For example, Nalla et al. collected serum samples from pregnant (early and late pregnancy) and non-pregnant women [42]. Subsequently, the effect of the sample administration was examined on rat neonatal β-cells and the rat insulinoma cell line. The most potent mitogenic effect was observed in samples exposed to late pregnancy sera. Isolated proliferative fractions contained PL, kininogen-1, fibrinogen-α-chain, α1-antitrypsin, apolipoprotein-A1, angiotensinogen, and serum albumin. Furthermore, the authors also discovered that the fractions had an inhibiting effect on insulinoma cell proliferation, suggesting that the activity of those factors could significantly contribute to the regulation of metabolic adaptations throughout pregnancy [42].

**Figure 1:**
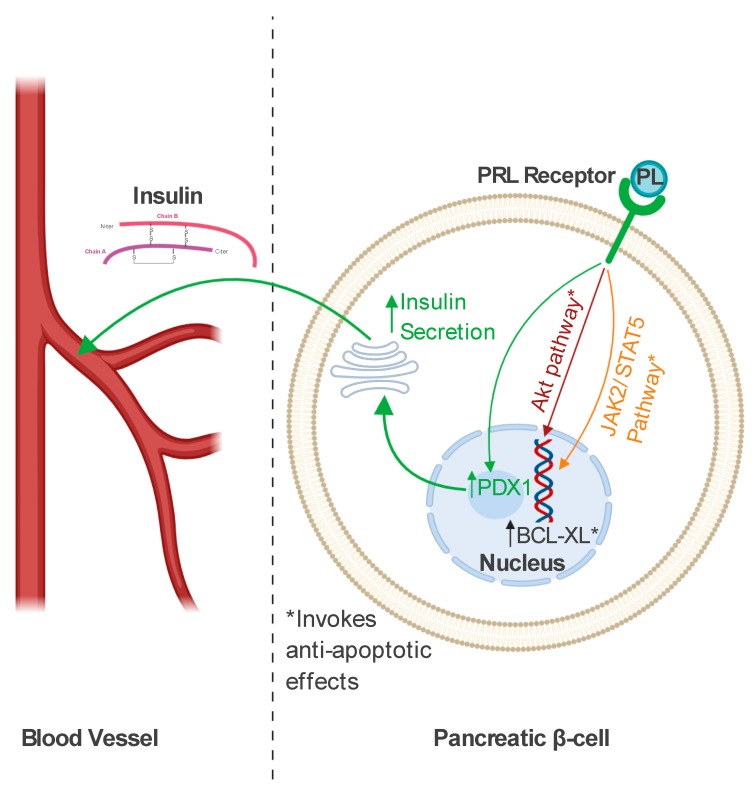
The mechanisms of the biological activity of placental lactogen (PL) in pancreatic β-cells. PL binds to the prolactin receptor (PRL Receptor) to promote increased insulin secretion through the stimulation of pancreatic and duodenal homeobox 1 (PDX1) expression. PL also activates a range of intracellular pathways (the Janus-activated-kinase-2/signal transducer and activator of transcription-5 (JAK2/STAT5) pathway and the phosphorylation of protein kinase B (AKT)) to protect β-cells from apoptotic death. Independently from the mechanisms mentioned above, PL contributes to increased expression of the BCL-XL anti-apoptotic protein [43,44,45,46]. Created with BioRender.

PL not only promotes β-cell proliferation but can also effectively inhibit apoptotic activity in murine and rat insulinoma cell lines through the phosphorylation of protein kinase B (AKT), as shown in Figure 1. The anti-apoptotic effect of PL was also observed in human pancreatic islet cells in vitro [43]. Cultured islets, treated with PL solution, secreted increased amounts of pancreatic and duodenal homeobox 1 (PDX1), which is an essential factor in pancreatic development [44]. The treatment resulted in improved glucose-induced insulin secretion compared with unstimulated control cells [43]. Furthermore, it has been reported that lactogens could protect pancreatic cells against glucolipotoxicity, which normally leads to beta cell death [45]. The next protective mechanism of lactogens was demonstrated in vitro in rat insulinoma cells and primary mouse beta cells exposed to prolactin treatment with the presence of dexamethasone, which is recognized as a beta cell apoptotic inducer. Beta cell death, mediated by exposure to dexamethasone, was significantly reduced in cell cultures treated with PRL. The reduction in beta cell deaths is believed to be related to the activity of the Janus-activated-kinase-2/signal transducer and activator of transcription-5 (JAK2/STAT5) pathway. Furthermore, lactogens participate in the expression of the Bcl-XL anti-apoptotic protein, the presence of which is required, independently of the JAK2/STAT5 pathway, to enhance their protective activity in rodent cells [46].

Finally, PL is responsible for the regulation of gestational adaptations of maternal pancreatic beta cells, which can prevent the development of glucose intolerance during pregnancy [40,41]. Moreover, it simultaneously acts as a stimulator of beta cell proliferation and has an anti-apoptotic effect on islet beta cells. However, it is important to mention that its proliferative effect on the population of human beta cells is less pronounced [40,41,42,43,45,46].

## 4. Clinical Utilities of Placental Lactogen

### 4.1. Maternal Applications: In Vitro and In Vivo Studies

#### 4.1.1. General Information

PL is believed to be involved in the regulation of both maternal and fetal gestational adaptation. However, the majority of PL is released into the maternal circulation. To compartmentalize the PL release into maternal and fetal circulation, Linnemann et al. used the dual in vitro perfusion of an isolated cotyledon, with PL concentrations measured in the perfusates and the placental tissue prior to and after perfusion. According to their results, only 0.05% of lactogen is transferred to the developing fetus, with the remaining percentage being released into the maternal circulation [47]. PL is also detectable in the amniotic fluid. However, its concentrations are generally lower compared with the maternal serum [48]. In a group of term newborns (40th week), those born via vaginal delivery had significantly lower levels of PL in both the umbilical vein and umbilical artery compared with those born through cesarean section [49]. PL blood concentrations in multiple pregnancies tend to rise compared with single pregnancies [50].

#### 4.1.2. Maternal Obesity and Food Intake

Maternal obesity is thought to have a disrupting effect on the expression of both members of the PL family hormones: PL and the placental growth hormone variant. A significant decrease in PL and *GH-V* RNA levels (75%) was observed in term placentas obtained from obese women (pre-pregnancy BMI > 35 kg/m^2^), compared to those with a normal BMI (BMI 20–25 kg/m^2^) [51]. These hormonal changes may be elucidated by the additionally detected downregulation of CCAAT enhancer-binding protein β (C/EBPβ) expression, which normally binds to the PL downstream enhancer and distally flanks the *GH-V* gene. Changes in C/EBPβ expression suppress PL and *GH-V* secretion [52]. Interestingly, in contrast to animal models, PL has been found to act as a potential stimulator of lipolysis during human pregnancy [53].

Leptin is one of the adipose-derived hormones. Leptin, as well as other adipocytokines, are produced and released from maternal adipocytes and syncytiotrophoblast cells during gestation [54]. It is thought that leptin, from the time of early pregnancy, plays an important role in placentation and the regulation of fetal growth. Furthermore, in later stages of pregnancy, leptin acts to decrease the maternal nutrient intake. However, to provide an optimal caloric consumption, pregnant women develop a state of central physiological leptin resistance [55,56]. Maternal obesity is closely linked with an excessive mass of adipose tissue and could potentially disrupt the regulation of the secretion of leptin and other adipocytokines [55]. PL has been found to have an inhibiting effect on leptin production in human cultured placental trophoblast cells, whereas, regardless of its blood concentration, leptin was unable to modify the production and secretion of PL [57,58]. However, its influence on leptin secretion in vivo and the possible effect on changes in the maternal food intake have not yet been studied.

Maternal obesity has been linked with decreased PL secretion [51,52]. Nonetheless, there is an insufficient number of studies focused on PL concentrations in obese mothers to speculate about its relationship with maternal obesity.

#### 4.1.3. Gestational and Pregestational Diabetes Mellitus

##### Molecular Aspects

The exposure of human pancreatic ductal cells (PANC-1) to a mixture of PL and human fibroblast growth factor-2b could effectively induce their de-differentiation to active islet β cells. After treatment, PANC-1 changed their ultrastructure to that typical of islets-aggregates, showing a significantly increased production of insulin and C peptide, which could be a promising new approach to diabetes treatment [59]. Similar to rodent models, PL stimulates the maternal prolactin receptors in human pancreatic β cells, promoting their adaptations to increased insulin requirements during physiological gestation [41,60]. In addition, some changes in the molecular structure of *PRLR* gene 5′ UTR and the promoter region were found to be associated with a 2.36-fold higher risk of gestational diabetes [60].

##### Placental Lactogen Concentrations throughout Pregnancy

Pregestational diabetes may be associated with increased blood levels of human PL, as shown in Table 1. Ngala et al. described elevated concentrations of first-trimester PL in a group of pregnant women with type 1 diabetes, in comparison to a healthy control group. Interestingly, they did not notice differences in PL blood levels between patients who developed gestational diabetes and healthy controls during the second trimester [61]. Other authors also did not find differences in PL blood levels among patients with GDM and without diabetes [62,63]. However, PL levels measured in the amniotic fluid in patients without diabetes were significantly lower compared with concentrations measured in patients with GDM [62]. Mills et al. have described similar findings, and also did not find any significant differences in PL mRNA levels among patients with GDM and without diabetes [64].

In contrast, different studies have found significant discrepancies in PL levels between patients with diabetes (detailed information about clinical characteristics—Table 1) and controls, suggesting that diabetic pregnancy could promote increased PL secretion [65,66,67]. More surprisingly, Botta et al. have consistently found significantly lower PL levels throughout pregnancy in patients with diabetes (White’s class B–C), compared with controls. The same study indicated that lower PL levels are inversely correlated with maternal blood glucose levels [68]. Moreover, there were not any significant correlations between PL levels and the measure of maternal insulin sensitivity/resistance, assessed using the Matsuda index or homeostatic model assessment of insulin resistance (HOMA-IR) [63]. Luthman et al. reported that plasma PL levels in pregnant patients were inversely correlated with the levels of pituitary growth hormone and growth hormone-binding protein (GHBP). Furthermore, they suggested that the elevation of PL might be responsible for a pituitary GH and GHBP decrease. However, it needs to be noted that this research involved a relatively small sample size [69].

Lopez-Espinoza et al. divided their study group into three smaller groups, including patients with insulin-dependent diabetes, gestational diabetes, and non-diabetic pregnant women. They found that the PL blood levels in all groups were positively correlated with urinary albumin excretion and the placental weight. Furthermore, the authors also found a positive correlation between PL and blood pressure, but only in the insulin-dependent group [70].

Analyzing the differences in PL concentrations in maternal blood throughout pregnancy, we cannot come to a definite conclusion about the influence of all types of diabetes on its secretion. This is mainly due to the fact that the vast majority of the studies were conducted in the past decades, when the diagnostic criteria of each type of diabetes were not clearly established. Moreover, in some studies, the authors did not define patients’ clinical characteristics (diabetes type), which could be regarded as a possible limitation of this review. Nevertheless, two studies with a relatively large number of participants (200, 395) showed no differences in PL levels between patients with GDM and healthy controls [61,63]. The situation in patients with pre-pregnancy diabetes looks different. We could speculate that worse glycemic control in patients with pregestational diabetes is associated with increased PL concentrations in maternal blood [61,65].

##### Placental Lactogen as a GDM Screening Tool

According to current recommendations of the American Diabetes Association, the screening for gestational diabetes is based on the results of a 75 g oral glucose tolerance test (OGTT), performed in women at 24–28 weeks of gestation, or on the alternative strategy of the “two-step” approach with a 50 g (non-fasting) screen. followed by a 100 g OGTT for those with positive screening results [71]. It has been reported that combining human PL measurements with routine oral glucose challenge tests could improve their positive predictive value in the diagnosis of gestational diabetes [72]. Patients’ glucose tolerance was initially assessed using the 1 h glucose challenge test (GCT), and those with positive results of GCT were subsequently examined using a 100 g OGTT. It has been found that patients with positive results for both GCT and OGTT have significantly higher blood concentrations of PL compared with those with positive GCT and negative OGTT results. Furthermore, 11 women from the group with normal GCT delivered infants that weighed > 4000 g. The average PL blood levels among them were similar to the mean PL levels determined in the group with positive OGTT and diagnosed diabetes [72].

Hence, the combination of oral glucose tests and PL measurement could contribute to the better detection of pregnancies with an increased risk of fetal macrosomia and other diabetes-related complications.

##### Diabetes Complications

It is believed that maternal diabetes mellitus could be one of the causative factors of placental villous immaturity due to the increased incidence of that pathology among patients with diabetes [73]. Placental villous immaturity may be associated with an increased risk of intrauterine fetal death, fetal growth restriction, and future chronic conditions [74]. Immunostaining performed on third-trimester diabetic placentas revealed decreased staining for PL compared with control placentas. Moreover, this pattern of staining was especially pronounced within the areas of marked architectural villous immaturity [75].

The state of acute maternal hypoglycemia is regarded as a life-threatening condition in most cases [76]. Björklund et al. induced hypoglycemia (glucose blood levels of about 2.2 mmol/L) in a group of insulin-dependent patients in the third trimester of pregnancy in an effort to understand the influence of hypoglycemia on placental hormones. PL blood levels did not change significantly, but there was a report of a significant elevation in placental growth hormone concentrations [77].

It has been observed that the increase in serum levels of placental somatolactogens (PL and GH-V) can be associated with the pathogenesis of diabetic retinopathy, with the mechanism of this process remaining unknown. Other somatolactogens, pituitary growth hormone, and prolactin act in this process as enhancers of angiogenesis, with the uncontrolled proliferation of new vessels related to the risk of vitreous hemorrhage and vision impairment [78,79].

Some authors have hypothesized that falling insulin requirements among patients with pregestational diabetes and GDM during late pregnancy are related to placental dysfunction, whereas others present no connection [80,81,82]. The exact influence of these findings on perinatal outcomes is still uncertain. Abnormalities in the production of placental hormones were nevertheless considered to be a potential causative factor of changes in insulin requirements, with the prospective observational study conducted by Padmanabhan et al. reporting no differences in PL, progesterone, and TNF-α blood concentrations in women with decreasing insulin requirements [83].

The secretion of IGF1 was found to be positively correlated with maternal blood levels of placental growth hormone rather than PL. The elevation in pituitary GH secretion in response to low plasma levels of placental GH was observed in patients with impairments in the functioning of the fetoplacental unit [84].

The possible influence of PL on the prevalence of diabetes complications during pregnancy has still not been well-established. There is no evidence that changes in PL levels can be associated with maternal hypoglycemia or the state of decreasing insulin requirements during late pregnancy, which is often associated with placental aging. Nonetheless, PL could somehow be involved in the pathogenesis of diabetic retinopathy and placental villous immaturity.

##### Long-Term Implications

Ratenkaren et al. suggested that the levels of antepartum lactogens could be related to glucose metabolism impairments after pregnancy. They found a possible positive effect of higher prolactin levels during gestation, with PL levels presenting no difference between the group of women with normal glucose tolerance and those with prediabetes or diabetes [85].

### 4.2. Fetal Growth and Perinatal Outcomes

#### 4.2.1. Association with Fetal Growth

To investigate the influence of human PL and somatomedin A on fetal growth, Kastrup et al. collected samples of maternal blood during the third trimester of pregnancy and cord blood at term. While somatomedin A was found to be associated with fetal growth parameters, PL did not correlate with fetal growth (birthweight and length) [26], as shown in Table 2.

In contrast, Knopp et al. discovered positive correlations between maternal PL and both birth weight and length [86]. Houghton et al. also found a correlation between term infant weight and maternal PL levels. However, cord PL levels were not associated with the birthweight of the newborn. Interestingly, elevated cord PL concentrations were revealed among female offspring [87]. Moreover, Männik et al. compared the placental expression of human growth factor and chorionic somatotropin gens in groups of small for gestational age (SGA), appropriate for gestational age (AGA), and large for gestational age (LGA) newborns. *CSH-1* and *CSH-2* genes are known to be positively correlated with blood PL concentrations. SGA and AGA neonates had significantly decreased levels of *CSH1-1* and *CSH2-1* mRNA expression compared with LGA newborns. Although *CSH1-1* and *CSH2-1* transcript levels were lower among SGA newborns, there was no significant difference between *CSH1-1* and *CSH2-1* in SGA and AGA infants [88]. A study performed in a group of 83 patients with insulin-dependent diabetes mellitus found that PL levels among women who delivered macrosomic newborns were significantly higher compared with the mothers of newborns with a normal weight [89]. This fact could suggest that PL is involved in the regulation of fetal growth, but fetal growth restriction tends to be more complex than macrosomia, for which a strong positive correlation was shown.

In summary, there is a sufficient amount of evidence to claim that PL is directly involved in the regulation of fetal growth (Table 2). Maternal blood levels of PL are significantly positively correlated with the fetal birth weight. 

#### 4.2.2. Human Placental Lactogen as a Gestational Marker of Fetal Development

High levels of maternal PL (highest quartile of results) measured at 18 weeks of gestation are associated with a significantly lower risk of fetal growth restriction (FGR) compared to lower quartiles. Furthermore, infants of mothers with the highest levels of PL, estradiol, and pregnancy-specific beta 1 glycoprotein had the lowest risk of FGR [90]. Markestad et al. measured the PL levels serially at 17, 25, 33, and 37 weeks of pregnancy. Overall, multiple studies present a correlation between the birth weight ratio (birth weight to mean birth weight for gestational age) and PL levels throughout gestation. The measurements of PL were found to be much more efficient in excluding growth retardation than predicting SGA [91].

Dutton et al. found a significant decrease in PL serum levels in women exhibiting reduced fetal movements and poor perinatal outcomes (SGA, preterm birth, neonatal intensive care unit admission after delivery). More interestingly, even though patients with poor perinatal outcomes had lower PL serum levels, there were not any discrepancies in PL mRNA expression in placental samples between them and those with normal perinatal outcomes [93].

Fetal crown-rump length (CRL) measurement in early pregnancy is regarded as an accurate diagnostic tool in the assessment of gestational age. However, it has also been established that PL could be used as a reliable marker for predicting gestational age during the first trimester. Gestational age estimated from PL measurements did not significantly differ from the results obtained from CRL measurements and the date of the last menstrual period in groups of patients with an uncomplicated pregnancy and those with insulin-dependent diabetes [94].

Pedersen et al. studied how differences in PL and pregnancy-associated plasma protein A measurements during early pregnancy could contribute to perinatal outcomes. A negative correlation with gestational age at birth occurred in both cases (PL and protein A), suggesting that higher PL levels during the first trimester result in better fetal growth throughout pregnancy. The authors speculated that earlier deliveries in patients with higher PL levels could be recognized as a consequence of better fetal development related to increased PL secretion [92]. However, these results are inconsistent with the hypothesis that PL could be used as a reliable marker of gestational age during the first trimester [94]. An accurate marker of gestational age during the first trimester should be independent of the other factors in the general population, and its levels cannot be used in predicting future intrauterine growth or birth weight.

**Figure 2:**
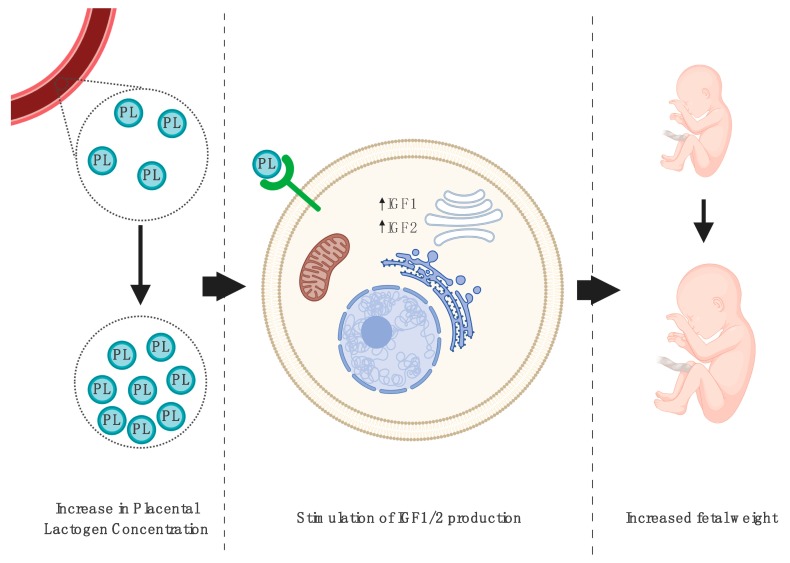
The influence of increased blood placental lactogen (PL) levels on tissues of the developing fetus. Elevated PL concentrations throughout pregnancy are correlated with an increased secretion of insulin-like growth factor-1 (IGF-1) and insulin-like growth factor-2 (IGF-2), which directly corresponds to an increased fetal weight [95]. Created with BioRender.

Lassarre et al. investigated the concentrations of IGF1, IGF2, IGF-binding protein, and PL. Fetal blood samples were collected using the method of percutaneous umbilical cord blood sampling performed between 20 and 37 weeks of gestation. The authors found significant positive correlations between IGF1, IGF2, and PL levels in samples obtained after 33 weeks of gestation, supporting the hypothesis that PL stimulates IGF production and plays a role in the regulation of fetal growth [95], as shown in Figure 2. Finally, when the fetal weight was only correlated with IGF1 levels, its concentrations were significantly higher in the group of newborns with a gestational weight higher than the mean and reduced in those with intrauterine growth restriction [95].

The measurement of PL can be used in the assessment of the risk of fetal growth restriction. However, regular ultrasonography seems to be more efficient. Furthermore, PL measures could allegedly give us information about the estimated fetal age during the first trimester, which could be useful in the situation when ultrasonography and CRL measurement are unavailable; however, these findings should be verified in further studies.

#### 4.2.3. How Could Maternal Malnutrition Affect Fetal Development and PL Secretion?

**Figure 3:**
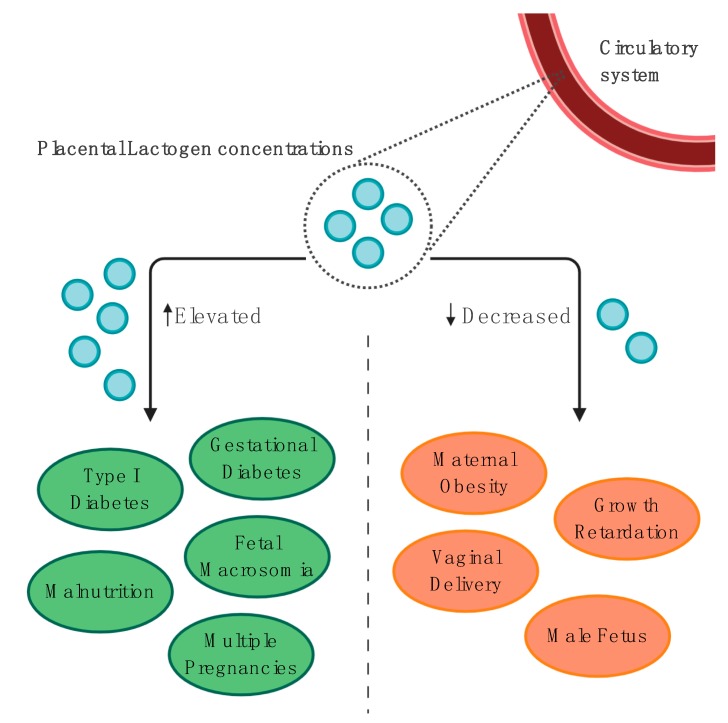
The summary of clinical conditions associated with elevated and decreased placental lactogen blood levels [49,50,51,61,65,66,67,86,87,88,89,90,91,92,99]. Created with BioRender.

Malnutrition among women is known to be one of the major healthcare issues in developing countries [96]. Proper nutrition is fundamental to good maternal health, especially during the second and third trimester of gestation to compensate for the increased energy requirements [97]. Newborns of malnourished mothers are generally more often smaller for gestational age, compared with those with an optimal state of nutrition [98]. Maternal malnutrition, along with anemia, contributes to the occurrence of adaptive changes in fetal and placental hormone secretion patterns. Surprisingly, SGA fetuses of mothers who suffered from malnutrition produced higher amounts of proteins such as GH, PRL, PL, and IGF1 [99], which likely occurs in response to the state of maternal malnutrition, as shown in Figure 3. Trophoblast cells stimulate fetal development by producing additional amounts of PL family proteins, but without sufficient nutrition, there is an elevated risk of developing intrauterine growth restriction. Furthermore, maternal PL levels tend to significantly rise in the state of acute starvation [100].

#### 4.2.4. Additional Contributions to the Regulation of Fetal Growth

In both humans and sheep, a single course of betamethasone was reported to be connected with fetal growth restriction. A decline in sheep PL, which disrupted fetal intrauterine growth, was found to be associated with a single steroid course. In contrast, betamethasone administration in humans did not alter maternal PL concentrations 48 h after treatment and plasma levels at birth [101,102]. Nonetheless, it is important to consider the fact that steroid therapy is often administered in patients at risk of preterm labor to stimulate fetal lung maturity, placing these pregnancies at risk of multifactorial growth restriction. Therefore, it is important to adjust the analysis to account for other potential growth restricting factors to fully consider the impact of betamethasone on perinatal growth.

The *PHLDA2* gene has been linked to the regulation of fetal growth and PL secretion in a mouse model. To examine its potential role in patients who experienced reduced fetal movements, the samples of placental tissue were obtained immediately after delivery. Afterwards, the expression of *PHLDA2* in collected placentas was determined. *PHLDA2* expression was found to be 2-3-fold higher in women with fetal growth restriction. Moreover, blood PL concentrations were negatively correlated with the levels of *PHLDA2* expression [103], suggesting that fetal growth restriction caused by inappropriate PL levels could be determined by increased *PHLDA2* gene expression.

## 5. Conclusions

Undoubtedly, both diabetes and obesity can interrupt the physiological synthesis and biological activity of placental lactogen in either humans or animal models. The role of obesity in the regulation of PL activity requires further investigation. However, it is thought that obesity promotes a deficiency in PL levels, which results in a range of metabolic disruptions. Both gestational and pregestational diabetes are said to be associated with several pregnancy complications, and their pathogenesis can be associated with alterations in PL production. The protective effect of placental lactogen on pancreatic islets is well-defined and could be used in new diabetes treatment strategies. An appropriate secretion pattern of PL, without a doubt, plays a significant role in the regulation of fetal and placental development. It has been reported that an assessment of the PL concentration could be useful in the prediction of fetal macrosomia in women with normal OGTT results; however, its utility as a screening tool in that context is not yet well-established. Moreover, its usefulness in evaluating the risk of fetal growth restriction or in the assessment of the gestational age during the first trimester can be regarded as interesting research findings, but its application in clinical practice seems to be limited in the era of ultrasonography.

## Figures and Tables

**Table 1:** Placental lactogen throughout diabetic pregnancy.

Study Title [Reference]	Number of Participants	Clinical Characteristics	Placental Lactogen Measurement, Method	Main Findings	First Author (Year of Study)
Placental peptides metabolism and maternal factors as predictors of risk of gestational diabetes in pregnant women. A case-control study. [61]	200	150 healthy participants—12 developed GDM, 50 controls with T1D	I and II trimester of pregnancy, Sandwich Elisa in maternal serum	I trimester: PL levels significantly higher in patients with T1D, II trimester: No difference in PL between 12 patients who developed GDM and 138 healthy controls, PL levels were significantly higher in patients with T1D compared with those with GDM	Ngala et al. (2017)
Maternal serum and amniotic fluid levels of human placental lactogen in gestational diabetes. [62]	46	16 patients with GDM, 30 healthy controls	Between the 37th and 39th weeks of gestation, radioimmunoassay in maternal serum and amniotic fluid	No difference in PL serum levels, significantly higher concentrations of amniotic fluid PL in patients with diabetes	Lolis et al. (1978)
Evaluation of circulating determinants of beta-cell function in women with and without gestational diabetes. [63]	395	105 patients with GDM, 290 healthy controls	PL was measured “in the late second trimester”, ELISA #20-HPLHU-E01 (Alpco) in maternal serum	No differences in PL levels between diabetic and non-patients with diabetes	Retnakaran et al. (2016)
Serial determinations of human placental lactogen in the management of diabetic pregnancy. [65]	138	98 patients with diabetes—White classes: A—14, B—36, C—28, D + F—20, 40 normal controls	PL concentrations were determined at the 27–29, 30–31, 32–34, and 35–37 weeks of gestation. Radioimmunoassay in maternal serum	PL concentrations after 32 weeks’ gestation were significantly higher in patients with diabetes compared with controls. No differences in PL levels between various White classes	Soler et al. (1975)
Placental Lactogen Levels in Diabetic Pregnancy. [66]	34	“patients with abnormal glucose tolerance tests during pregnancy”, 29 insulin-dependent	A total of 219 measurements in 34 patients. At each visit from 20 weeks’ gestation, and weekly from 32 weeks until delivery. Radioimmunoassay in maternal serum	PL serum levels higher than those in a normal population	Ursell et al. (1973)
Mitochondrial function and glucose metabolism in the placenta with gestational diabetes mellitus: Role of miR-143. [67]	18	6 patients with A1GDM, 6 patients with A2GDM, 6 patients that were healthy controls	Placental tissue collected at term after C-section, Sandwich Elisa (Genway)—in placental homogenate	PL significantly increased in A2GDM patients compared with those with A1GDM and controls	Muralimanoharan et al. (2016)
Placental lactogen, progesterone, total estriol and prolactin plasma levels in pregnant women with insulin-dependent diabetes mellitus. [68]	25	15 insulin-dependent patients (White’s class B–C), 10 healthy controls	PL measured every 4 weeks from the 12th to 36th week of gestation. Radioimmunoassay in maternal serum (Biodata kit)	PL significantly lower in patients with diabetes at the 12th, 20th, 24th, 32nd, and 36th weeks of gestation	Botta et al. (1984)

Abbreviations: A1GDM—gestational diabetes controlled by diet and exercise, A2GDM—gestational diabetes treated with medication, GDM—gestational diabetes mellitus, PL—placental lactogen, and T1D—type 1 diabetes.

**Table 2:** Placental lactogen in the regulation of fetal growth.

Study Title [Reference]	Clinical Characteristics	Analyzed Parameters	Main Findings	First Author (Year of Study)
Somatomedin in newborns and the relationship to human chorionic somatotropin and fetal growth. [26]	22 pregnant patients	PL levels in the maternal serum during the III trimester of pregnancy and cord blood at term	No correlation between PL levels, and birth weight and length	Kastrup et al. (1978)
Relationships of infant birth size to maternal lipoproteins, apoproteins, fuels, hormones, clinical chemistries, and body weight at 36 weeks gestation. [86]	273 patients in singelton pregnancies	PL concentrations in maternal blood measured at 36 weeks of gestation	Positive correlation between maternal blood PL concentrations, birth weight, and birth length	Knopp et al. (1985)
Relationship of maternal and fetal levels of human placental lactogen to the weight and sex of the fetus. [87]	101 pregnant patients	PL levels in the maternal serum at 38–42 weeks of gestation, cord artery, and cord vein collected at term	Positive correlation between maternal serum PL and birth weight, with no correlation in the case of umbilical cord blood	Houghton et al. (1984)
Differential expression profile of Growth Hormone/Chorionic Somatomammotropin genes in placenta of small- and large-for-gestational-age newborns. [88]	72 patients in uncomplicated singelton pregnancies	CSH1 and CSH2 gene mRNA in term placental tissue	CSH1 and CSH2 gene transcript levels were significantly higher in LGA newborns compared with SGA and AGA neonates	Männik et al. (2010)
Macrosomia in Pregnancy Complicated by Insulin-Dependent Diabetes Mellitus. [89]	83 patients with insulin-dependent diabetes	PL maternal serum concentrations during the III trimester of pregnancy	Mothers of macrosomic infants have significantly higher concentrations of serum PL	Small et al. (1987)
Maternal serum concentrations of human placental lactogen, estradiol and pregnancy specific β1-glycoprotein and fetal growth retardation. [90]	200 multiparous women with fetal growth retardation risk factors	PL maternal serum levels measured at a mean of 18 weeks’ gestational age	Higher maternal levels of PL are associated with a decreased prevalence of fetal growth retardation	Gardner (1997)
Prediction of fetal growth based on maternal serum concentrations of human chorionic gonadotropin, human placental lactogen and estriol. [91]	214 patients, mothers of 102 SGA infants and 112 non-SGA neonates	PL levels in maternal serum were measured serially at 17, 25, 33, and 37 weeks of gestation	Significant differences in PL measured at 17, 33, and 37 weeks of pregnancy in mothers of SGA and non-SGA infants	Markestad et al. (1997)
Human placental lactogen and pregnancy-associated plasma protein A in first trimester and subsequent fetal growth. [92]	93 patients with uncomplicated singelton pregnancies	Maternal PL serum concentrations measured between the 8th and 14th week of pregnancy	PL is negatively correlated with gestational age at delivery	Pedersen et al. (1995)

Abbreviations: AGA—appropriate for gestational age, CSH1—chorionic somatomammotropin 1, CSH2—chorionic somatomammotropin 2, GDM—gestational diabetes mellitus, LGA—large for gestational age, PL—placental lactogen, and SGA—small for gestational age.

## References

[1] Hales C.M., Carroll M.D., Fryar C.D., Ogden C.L. (2017). Prevalence of Obesity Among Adults and Youth: United States, 2015-2016. NCHS Data Brief.

[2] Haslam D.W., James W.P.T. (2005). Obesity. Lancet.

[3] Guh D.P., Zhang W., Bansback N., Amarsi Z., Birmingham C.L., Anis A.H. (2009). The incidence of co-morbidities related to obesity and overweight: A systematic review and meta-analysis. BMC Public Health.

[4] Catalano P.M., Shankar K. (2017). Obesity and pregnancy: Mechanisms of short term and long term adverse consequences for mother and child. BMJ.

[5] Huda S.S., Brodie L.E., Sattar N. (2010). Obesity in pregnancy: Prevalence and metabolic consequences. Semin. Fetal Neonatal Med..

[6] Vasudevan C., Renfrew M., McGuire W. (2011). Fetal and perinatal consequences of maternal obesity. Arch. Dis. Child. Fetal Neonatal Ed..

[7] Garne E., Loane M., Dolk H., Barisic I., Addor M.C., Arriola L., Bakker M., Calzolari E., Matias Dias C., Doray B. (2012). Spectrum of congenital anomalies in pregnancies with pregestational diabetes. Birth Defects Res. Part A Clin. Mol. Teratol..

[8] Ali S., Dornhorst A. (2011). Diabetes in pregnancy: Health risks and management. Postgrad. Med. J..

[9] Vambergue A., Fajardy I. (2011). Consequences of gestational and pregestational diabetes on placental function and birth weight. World J. Diabetes.

[10] Wender-Ozegowska E., Bomba-Opoń D., Brazert J., Celewicz Z., Czajkowski K., Gutaj P., Malinowska-Polubiec A., Zawiejska A., Wielgoś M. (2018). Standards of Polish Society of Gynecologists and Obstetricians in management of women with diabetes. Ginekol. Pol..

[11] Bernasko J. (2007). Intensive insulin therapy in pregnancy: Strategies for successful implementation in pregestational diabetes mellitus. J. Matern. Neonatal Med..

[12] Miller W.L., Eberhardt N.L. (1983). Structure and evolution of the growth hormone gene family. Endocr. Rev..

[13] Hill D.J. (2018). Placental control of metabolic adaptations in the mother for an optimal pregnancy outcome. What goes wrong in gestational diabetes?. Placenta.

[14] Faria T.N., Deb S., Kwok S.C.M., Talamantes F., Soares M.J. (1990). Ontogeny of placental lactogen-I and placental lactogen-II expression in the developing rat placenta. Dev. Biol..

[15] Yamaguchi M., Ogren L., Endo H., Thordarson G., Bigsby R.M., Talamantes F. (1992). Production of mouse placental lactogen-I and placental lactogen-II by the same giant cell. Endocrinology.

[16] Lacroix M.C., Bolifraud P., Durieux D., Pauloin A., Vidaud M., Kann G. (2002). Placental growth hormone and lactogen production by perifused ovine placental explants: Regulation by growth hormone-releasing hormone and glucose. Biol. Reprod..

[17] Alvarez-Oxiley A.V., de Sousa N.M., Beckers J.-F. (2008). Native and recombinant bovine placental lactogens. Reprod. Biol..

[18] Walker W.H., Fitzpatrick S.L., Saunders G.F., Barrera-Saldaña H.A., Resendez-Perez D. (1991). The human placental lactogen genes: Structure, function, evolution and transcriptional regulation. Endocr. Rev..

[19] Handwerger S. (1991). Clinical counterpoint: The physiology of placental lactogen in human pregnancy. Endocr. Rev..

[20] Untergasser G., Hermann M., Rumpold H., Pfister G., Berger P. (2000). An unusual member of the human growth hormone/placental lactogen (GH/PL) family, the testicular alternative splicing variant hPL-A2: Recombinant expression revealed a membrane-associated growth factor molecule. Mol. Cell. Endocrinol..

[21] Handwerger S., Freemark M. (2000). The roles of placental growth hormone and placental lactogen in the regulation of human fetal growth and development. J. Pediatr. Endocrinol. Metab..

[22] Hill D.J., Freemark M., Strain A.J., Handwerger S., Milner R.D.G. (1988). Placental lactogen and growth hormone receptors in human fetal tissues: Relationship to fetal plasma human placental lactogen concentrations and fetal growth. J. Clin. Endocrinol. Metab..

[23] Colosi P., Talamantes F., Linzer D.I.H. (1987). Molecular cloning and expression of mouse placental lactogen i complementary deoxyribonucleic acid. Mol. Endocrinol..

[24] Colosi P., Marr G., Lopez J., Haro L., Ogren L., Talamantes F. (1982). Isolation, purification, and characterization of mouse placental lactogen. Proc. Natl. Acad. Sci. USA.

[25] Harigaya T., Smith W.C., Talamantes F. (1988). Hepatic placental lactogen receptors during pregnancy in the mouse. Endocrinology.

[26] Kastrup K.W., Andersen H.J., Lebech P. (1978). Somatomedin in newborns and the relationship to human chorionic somatotropin and fetal growth. Acta Pædiatrica.

[27] Newbern D., Freemark M. (2011). Placental hormones and the control of maternal metabolism and fetal growth. Curr. Opin. Endocrinol. Diabetes Obes..

[28] Ryan E.A., Enns L. (1988). Role of gestational hormones in the induction of insulin resistance. J. Clin. Endocrinol. Metab..

[29] Barbour L.A., Shao J., Qiao L., Pulawa L.K., Jensen D.R., Bartke A., Garrity M., Draznin B., Friedman J.E. (2002). Human placental growth hormone causes severe insulin resistance in transgenic mice. Am. J. Obstet. Gynecol..

[30] Ladyman S.R., Augustine R.A., Grattan D.R. (2010). Hormone interactions regulating energy balance during pregnancy. J. Neuroendocrinol..

[31] Baker C.M., Goetzmann L.N., Cantlon J.D., Jeckel K.M., Winger Q.A., Anthony R.V. (2016). Development of ovine chorionic somatomammotropin hormone-deficient pregnancies. Am. J. Physiol. Regul. Integr. Comp. Physiol..

[32] Jeckel K.M., Boyarko A.C., Bouma G.J., Winger Q.A., Anthony R.V. (2018). Chorionic somatomammotropin impacts early fetal growth and placental gene expression. J. Endocrinol..

[33] Karabulut A.K., Layfield R., Pratten M.K. (2001). Growth promoting effects of human placental lactogen during early organogenesis: A link to insulin-like growth factors. J. Anat..

[34] Fleenor D., Oden J., Kelly P.A., Mohan S., Alliouachene S., Pende M., Wentz S., Kerr J., Freemark M. (2005). Roles of the lactogens and somatogens in perinatal and postnatal metabolism and growth: Studies of a novel mouse model combining lactogen resistance and growth hormone deficiency. Endocrinology.

[35] Arumugam R., Fleenor D., Freemark M. (2007). Effects of lactogen resistance and GH deficiency on mouse metabolism: Pancreatic hormones, adipocytokines, and expression of adiponectin and insulin receptors: Lactogen resistance and GH deficiency in mice. Endocrine.

[36] Fielder P.J., Talamantes F. (1992). The insulin-like effects of mouse growth hormone on adipose tissue from virgin and pregnant mice. Metab. Clin. Exp..

[37] Leturque A., Hauguel S., Sutter Dub M.T., Maulard P., Girard J. (1989). Effects of placental lactogen and progesterone on insulin stimulated glucose metabolism in rat muscles in vitro. Diabete Metab..

[38] Houseknecht K.L., Bauman D.E., Vernon R.G., Byatt J.C., Collier R.J. (1996). Insulin-like growth factors-I and -II, somatotropin, prolactin, and placental lactogen are not acute effectors of lipolysis in ruminants. Domest. Anim. Endocrinol..

[39] Campbell R.M., Kostyo J.L., Scanes C.G. (1990). Lipolytic and antilipolytic effects of human growth hormone, its 20-kilodalton variant, a reduced and carboxymethylated derivative, and human placental lactogen on chicken adipose tissue in vitro. Proc. Soc. Exp. Biol. Med..

[40] Chen H., Kleinberger J.W., Takane K.K., Salim F., Fiaschi-Taesch N., Pappas K., Parsons R., Jiang J., Zhang Y., Liu H. (2015). Augmented STAT5 signaling bypasses multiple impediments to lactogen-mediated proliferation in human β-cells. Diabetes.

[41] Banerjee R.R., Cyphert H.A., Walker E.M., Chakravarthy H., Peiris H., Gu X., Liu Y., Conrad E., Goodrich L., Stein R.W. (2016). Gestational diabetes mellitus from inactivation of prolactin receptor and MafB in islet β-cells. Diabetes.

[42] Nalla A., Ringholm L., Søstrup B., Højrup P., Thim L., Levery S.B., Vakhrushev S.Y., Billestrup N., Mathiesen E.R., Damm P. (2014). Implications for the offspring of circulating factors involved in beta cell adaptation in pregnancy. Acta Obstet. Gynecol. Scand..

[43] Lombardo M.F., De Angelis F., Bova L., Bartolini B., Bertuzzi F., Nano R., Capuani B., Lauro R., Federici M., Lauro D. (2011). Human placental lactogen (hPL-A) activates signaling pathways linked to cell survival and improves insulin secretion in human pancreatic islets. Islets.

[44] Offield M.F., Jetton T.L., Labosky P.A., Ray M., Stein R.W., Magnuson M.A., Hogan B.L.M., Wright C.V.E. (1996). PDX-1 is required for pancreatic outgrowth and differentiation of the rostral duodenum. Development.

[45] Kondegowda N.G., Mozar A., Chin C., Otero A., Garcia-Ocaña A., Vasavada R.C. (2012). Lactogens protect rodent and human beta cells against glucolipotoxicity- induced cell death through Janus kinase-2 (JAK2)/signal transducer and activator of transcription-5 (STAT5) signalling. Diabetologia.

[46] Fujinaka Y., Takane K., Yamashita H., Vasavada R.C. (2007). Lactogens promote beta cell survival through JAK2/STAT5 activation and Bcl-XL upregulation. J. Biol. Chem..

[47] Linnemann K., Malek A., Sager R., Blum W.F., Schneider H., Fusch C. (2000). Leptin production and release in the dually in vitro perfused human placenta. J. Clin. Endocrinol. Metab..

[48] Sarandakou A., Kassanos D., Phocas I., Kontoravdis A., Chryssicopoulos A., Zourlas P.A. (1992). Amniotic fluid hormone profiles during normal and abnormal pregnancy. Clin. Exp. Obs. Gynecol..

[49] Hercz P., Siklos P., Ungár L., Farquharson R.G., Mohári K., Kocsár L. (1987). Change of serum HPL level in maternal vein, umbilical cord vein and artery in mature and premature labour. Eur. J. Obstet. Gynecol. Reprod. Biol..

[50] Lebech P.E., Borggaard B. (1974). Serum levels of human chorionic somatomammotropin (HCS) in normal and abnormal pregnancies. Acta Endocrinol..

[51] Jin Y., Vakili H., Yan Liu S., Menticoglou S., Bock M.E., Cattini P.A. (2018). Chromosomal architecture and placental expression of the human growth hormone gene family are targeted by pre-pregnancy maternal obesity. Am. J. Physiol. Endocrinol. Metab..

[52] Vakili H., Jin Y., Menticoglou S., Cattini P.A. (2013). CCAAT-enhancer-binding protein β (C/EBPβ) and downstream human placental growth hormone genes are targets for dysregulation in pregnancies complicated by maternal obesity. J. Biol. Chem..

[53] Williams C., Coltart T.M. (1978). Adipose tissue metabolism in pregnancy: The lipolytic effect of human placental lactogen. BJOG Int. J. Obstet. Gynaecol..

[54] Pérez-Pérez A., Toro A., Vilariño-García T., Maymó J., Guadix P., Dueñas J.L., Fernández-Sánchez M., Varone C., Sánchez-Margalet V. (2018). Leptin action in normal and pathological pregnancies. J. Cell. Mol. Med..

[55] Tessier D.R., Ferraro Z.M., Gruslin A. (2013). Role of leptin in pregnancy: Consequences of maternal obesity. Placenta.

[56] Page-Wilson G., Reitman-Ivashkov E., Meece K., White A., Rosenbaum M., Smiley R.M., Wardlaw S.L. (2013). Cerebrospinal fluid levels of leptin, proopiomelanocortin, and agouti-related protein in human pregnancy: Evidence for leptin resistance. J. Clin. Endocrinol. Metab..

[57] Coya R., Martul P., Algorta J., Aniel-Quiroga M.A., Busturia M.A., Señarís R. (2005). Progesterone and human placental lactogen inhibit leptin secretion on cultured trophoblast cells from human placentas at term. Gynecol. Endocrinol..

[58] Coya R., Martul P., Algorta J., Aniel-Quiroga M.A., Busturia M.A., Señarís R. (2006). Effect of leptin on the regulation of placental hormone secretion in cultured human placental cells. Gynecol. Endocrinol..

[59] Donadel G., Pastore D., Della-Morte D., Capuani B., Lombardo M.F., Pacifici F., Bugliani M., Grieco F.A., Marchetti P., Lauro D. (2017). FGF-2b and h-PL transform duct and non-endocrine human pancreatic cells into endocrine insulin secreting cells by modulating differentiating genes. Int. J. Mol. Sci..

[60] Le T.N., Elsea S.H., Romero R., Chaiworapongsa T., Francis G.L. (2013). Prolactin receptor gene polymorphisms are associated with gestational diabetes. Genet. Test. Mol. Biomarkers.

[61] Ngala R.A., Fondjo L.A., Gmagna P., Ghartey F.N., Awe M.A. (2017). Placental peptides metabolism and maternal factors as predictors of risk of gestational diabetes in pregnant women. A case-control study. PLoS ONE.

[62] Lolis D., Tzingounis V., Kaskarelis D. (1978). Maternal serum and amniotic fluid levels of human placental lactogen in gestational diabetes. Eur. J. Clin. Invest..

[63] Retnakaran R., Ye C., Kramer C.K., Connelly P.W., Hanley A.J., Sermer M., Zinman B. (2016). Evaluation of circulating determinants of beta-cell function in women with and without gestational diabetes. J. Clin. Endocrinol. Metab..

[64] Mills N.C., Gyves M.T., Ilan J. (1985). Comparisons of human placental lactogen mRNA levels from placentas of diabetics and normal term. Mol. Cell. Endocrinol..

[65] Soler N.G., Nicholson H.O., Malins J.M. (1975). Serial determinations of human placental lactogen in the management of diabetic pregnancy. Lancet.

[66] Ursell W., Brudenell M., Chard T. (1973). Placental Lactogen Levels in Diabetic Pregnancy. Br. Med. J..

[67] Muralimanoharan S., Maloyan A., Myatt L. (2016). Mitochondrial function and glucose metabolism in the placenta with gestational diabetes mellitus: Role of miR-143. Clin. Sci..

[68] Botta R.M., Donatelli M., Bucalo M.L., Bellomonte M.L., Bompiani G.D. (1984). Placental lactogen, progesterone, total estriol and prolactin plasma levels in pregnant women with insulin-dependent diabetes mellitus. Eur. J. Obstet. Gynecol. Reprod. Biol..

[69] Luthman M., Stock S., Werner S., Bremme K. (1994). Growth hormone-binding protein in plasma is inverselycorrelated to placental lactogen and augmented with increasing body mass index in healthy pregnant women and women with gestational diabetes mellitus. Gynecol. Obstet. Invest..

[70] Lopez-Espinoza I., Smith R.F., Gillmer M., Schidlmeir A., Hockaday T.D. (1986). High levels of growth hormone and human placental lactogen in pregnancy complicated by diabetes. Diabetes Res..

[71] American Diabetes Association (2019). Classification and diagnosis of diabetes: Standards of medical care in diabetesd2019. Diabetes Care.

[72] Henderson C.E., Divon M.Y. (1998). Combining human placental lactogen with routine glucose challenge tests. Prim. Care Update Ob. Gyns..

[73] Daskalakis G., Marinopoulos S., Krielesi V., Papapanagiotou A., Papantoniou N., Mesogitis S., Antsaklis A. (2008). Placental pathology in women with gestational diabetes. Acta Obstet. Gynecol. Scand..

[74] Redline R.W. (2012). Distal villous immaturity. Diagnostic Histopathol..

[75] Greco M.A., Kamat B.R., Demopoulos R.I. (1989). Placental protein distribution in maternal diabetes mellitus: An immunocytochemical study. Fetal Pediatr. Pathol..

[76] Persson B., Hansson U. (1993). Hypoglycaemia in pregnancy. Baillieres. Clin. Endocrinol. Metab..

[77] Björklund A.O., Adamson U.K.C., Carlström K.A.M., Hennen G., Igout A., Lins P.E.S., Westgren L.M.R. (1998). Placental hormones during induced hypoglycaemia in pregnant women with insulin-dependent diabetes mellitus: Evidence of an active role for placenta in hormonal counter-regulation. BJOG Int. J. Obstet. Gynaecol..

[78] Larinkari J., Laatikainen L., Ranta T., Mörönen P., Pesonen K., Laatikainen T. (1982). Metabolic control and serum hormone levels in relation to retinopathy in diabetic pregnancy. Diabetologia.

[79] Bermea K.C., Rodríguez-García A., Tsin A., Barrera-Saldaña H.A. (2018). Somatolactogens and diabetic retinopathy. Growth Horm. IGF Res..

[80] Holmes D. (2017). Falling insulin requirements—a red flag for pre-eclampsia. Nat. Rev. Endocrinol..

[81] Ram M., Feinmesser L., Shinar S., Maslovitz S. (2017). The importance of declining insulin requirements during pregnancy in patients with pre-gestational gestational diabetes mellitus. Eur. J. Obstet. Gynecol. Reprod. Biol..

[82] Padmanabhan S., McLean M., Cheung N.W. (2014). Falling insulin requirements are associated with adverse obstetric outcomes in women with preexisting diabetes. Diabetes Care.

[83] Padmanabhan S., Lee V.W., McLean M., Athayde N., Lanzarone V., Khoshnow Q., Peek M.J., Cheung N.W. (2017). The association of falling insulin requirements with maternal biomarkers and placental dysfunction: A prospective study of women with preexisting diabetes in pregnancy. Diabetes Care.

[84] Caufriez A., Frankenne F., Hennen G., Copinschi G. (1993). Regulation of maternal IGF-I by placental GH in normal and abnormal human pregnancies. Am. J. Physiol. Endocrinol. Metab..

[85] Retnakaran R., Ye C., Kramer C.K., Connelly P.W., Hanley A.J., Sermer M., Zinman B. (2016). Maternal serum prolactin and prediction of postpartum b-cell function and risk of prediabetes/diabetes. Diabetes Care.

[86] Knopp R.H., Bergelin R.O., Wahl P.W., Walden C.E. (1985). Relationships of infant birth size to maternal lipoproteins, apoproteins, fuels, hormones, clinical chemistries, and body weight at 36 weeks gestation. Diabetes.

[87] Houghton D.J., Shackleton P., Obiekwe B.C., Chard T. (1984). Relationship of maternal and fetal levels of human placental lactogen to the weight and sex of the fetus. Placenta.

[88] Männik J., Vaas P., Rull K., Teesalu P., Rebane T., Laan M. (2010). Differential expression profile of Growth Hormone/Chorionic Somatomammotropin genes in placenta of small- and large-for-gestational-age newborns. J. Clin. Endocrinol. Metab..

[89] Small M., Cameron A., Lunan C.B., MacCuish A.C. (1987). Macrosomia in Pregnancy Complicated by Insulin-Dependent Diabetes Mellitus. Diabetes Care.

[90] Gardner M.O. (1997). Maternal serum concentrations of human placental lactogen, estradiol and pregnancy specific β1-glycoprotein and fetal growth retardation. Acta Obstet. Gynecol. Scand. Suppl..

[91] Markestad T. (1997). Prediction of fetal growth based on maternal serum concentrations of human chorionic gonadotropin, human placental lactogen and estriol. Acta Obstet. Gynecol. Scand. Suppl..

[92] Pedersen J.F., Sørensen S., Ruge S. (1995). Human placental lactogen and pregnancy-associated plasma protein A in first trimester and subsequent fetal growth. Acta Obstet. Gynecol. Scand..

[93] Dutton P.J., Warrander L.K., Roberts S.A., Bernatavicius G., Byrd L.M., Gaze D., Kroll J., Jones R.L., Sibley C.P., Frøen J.F. (2012). Predictors of poor perinatal outcome following maternal perception of reduced fetal movements—A prospective cohort study. PLoS ONE.

[94] Whittaker P.G., Aspillaga M.O., Lind T. (1983). Accurate assessment of early gestational age in normal and diabetic women by serum human placental lactogen concentration. Lancet.

[95] Lassarre C., Hardouin S., Daffos F., Forestier F., Frankenne F., Binoux M. (1991). Serum insulin-like growth factors and insulin-like growth factor binding proteins in the human fetus. Relationships with growth in normal subjects and in subjects with intrauterine growth retardation. Pediatr. Res..

[96] Müller O., Krawinkel M. (2005). Malnutrition and health in developing countries. CMAJ.

[97] Kominiarek M.A., Rajan P. (2016). Nutrition Recommendations in Pregnancy and Lactation. Med. Clin. North Am..

[98] Belkacemi L., Nelson D.M., Desai M., Ross M.G. (2010). Maternal undernutrition influences placental-fetal development. Biol. Reprod..

[99] Mahajan S.D., Singh S., Shah P., Gupta N., Kochupillai N. (2004). Effect of maternal malnutrition and anemia on the endocrine regulation of fetal growth. Endocr. Res..

[100] Tyson J.E., Austin K., Farinholt J., Fiedler J. (1976). Endocrine-metabolic response to acute starvation in human gestation. Am. J. Obstet. Gynecol..

[101] Braun T., Husar A., Challis J.R.G., Dudenhausen J.W., Henrich W., Plagemann A., Sloboda D.M. (2013). Growth restricting effects of a single course of antenatal betamethasone treatment and the role of human placental lactogen. Placenta.

[102] Braun T., Li S., Moss T.J.M., Newnham J.P., Challis J.R.G., Gluckman P.D., Sloboda D.M. (2007). Maternal betamethasone administration reduces binucleate cell number and placental lactogen in sheep. J. Endocrinol..

[103] Janssen A.B., Tunster S.J., Heazell A.E.P., John R.M. (2016). Placental PHLDA2 expression is increased in cases of fetal growth restriction following reduced fetal movements. BMC Med. Genet..

